# Honey and Diabetes: The Importance of Natural Simple Sugars in Diet for Preventing and Treating Different Type of Diabetes

**DOI:** 10.1155/2018/4757893

**Published:** 2018-02-04

**Authors:** Otilia Bobiş, Daniel S. Dezmirean, Adela Ramona Moise

**Affiliations:** ^1^Life Sciences Institute “King Michael I of Romania”, University of Agricultural Sciences and Veterinary Medicine, Cluj-Napoca, Romania; ^2^Faculty of Animal Breeding and Biotechnology, Technology Department, University of Agricultural Sciences and Veterinary Medicine, Cluj-Napoca, Romania

## Abstract

Diabetes is a metabolic disorder with multifactorial and heterogeneous etiologies. Two types of diabetes are common among humans: type 1 diabetes that occurs when the immune system attacks and destroys insulin and type 2 diabetes, the most common form, that may be caused by several factors, the most important being lifestyle, but also may be determined by different genes. Honey was used in folk medicine for a long time, but the health benefits were explained in the last decades, when the scientific world was concerned in testing and thus explaining the benefits of honey. Different studies demonstrate the hypoglycemic effect of honey, but the mechanism of this effect remains unclear. This review presents the experimental studies completed in the recent years, which support honey as a novel antidiabetic agent that might be of potential significance for the management of diabetes and its complications and also highlights the potential impacts and future perspectives on the use of honey as an antidiabetic agent.

## 1. Introduction

Diabetes mellitus is one of the top diseases in modern times, with more than 285 million people estimated in 2010 and about 438 million people predicted for 2030 in all over the world [1]. Diabetes prevalence may be genetically determined or can be developed during lifetime at any age. This disease takes no account of age for example, but scientific studies reveal that it is more common in developing countries than in the rest of the world (developed countries and third world countries) [1]. The increasing incidence may be due to demographic changes and undesirable result of risk factors such as obesity and sedentary life.

What is in fact diabetes mellitus? Diabetes is a metabolic disorder with multifactorial and heterogeneous etiologies. The high blood sugar level is the “symptom” known for diabetes, but other symptoms should not be ignored: increased thirst and hunger, unexplained fatigue, increased urination, blurred vision, and unexpected weight loss. Two types of diabetes are common among humans: type 1 diabetes that occurs when the immune system attacks and destroys insulin. This type of diabetes is believed to be genetically determined but also environmental factors are important in the determination of the disease. The symptoms of this type of diabetes generally start quickly, in a matter of weeks. Type 2 diabetes, the most common form, may be caused by several factors, the most important being lifestyle, but also it may be determined by different genes. This type of disease is developed during several years, and the symptoms are also not noticeable; for this reason, many people find themselves with diabetes without specific or unusual symptoms. Type 2 diabetes is most of the time related to overweight or obese state.

Although diabetes mellitus is a chronic disease of endocrine diagnosis and remains the major cause of mortality worldwide [2–5], it is not a death sentence.

Nowadays, the medical world is turning more and more on the health benefits of natural products, medicinal herbs, and also honey, in the management of this illness. Together with classic medical treatment, using recipes of traditional medicine, including the use of apicultural products (i.e., honey), the diabetic patients can maintain the normal level of insulin in the blood and also their overall health condition.

Honey composition comprises more than 200 components, with fructose, glucose, and water as main substances. Honey was used in folk medicine back in time at the beginning of our era, but their health benefits were based only on eye observations, without having any basis for scientific support. Only in the last decades, the scientific world was concerned in testing and explaining the benefits of honey. These research studies explain to a large extent many medicinal effects of honey such as antioxidant [6–11], hepatoprotective [12–14], cardioprotective [15–17], antibacterial [18–23], anti-inflammatory [24–26], or antitumor [27–30].

For a long time, there has been a myth that honey could not be used in diabetic patient's diet, due to the high content of carbohydrates from its chemical composition. Considering the background of the research team that has been working on characterization of different types of honey from Romania and worldwide and the determination of its biological properties for a long period, we considered being appropriate to gather in a review, literature studies that may answer the question: is honey a good substitute for sugar in diabetic diet? Are natural simple sugars important in preventing and treating diabetes mellitus?

Therefore, the present study acknowledged different scientific studies, demonstrating the use of honey in diabetes mellitus: preclinical and clinical studies, animal model studies, and human studies that demonstrate the potential impact of honey on this complex disease.

## 2. Fructose and the Hypoglycemic Effect of Honey

Fructose content of honey varies from 21 to 43% and the fructose/glucose ratio from 0.4 to 1.6 or even higher [31–34]. Although fructose is the sweetest naturally occurring sweetener, it has a glycemic index of 19, compared to glucose which has 100 or sucrose (refined sugar) with 60 [35]. Different studies reveal the hypoglycemic effect of honey, but the mechanism of this effect remains unclear. It was suggested that fructose, selective mineral ions (selenium, zinc, copper, and vanadium), phenolic acids, and flavonoids might have a role in the process [10, 11, 31, 33, 36, 37].

There is evidence that fructose tends to lower blood glucose in animal models of diabetes [38, 39]. Mechanisms involved in this process may include reduced rate of intestinal absorption [40], prolongation of gastric emptying time [41, 42], and reduced food intake [43, 44]. Fructose stimulates glucokinase in hepatocytes, which plays an important role in the uptake and storage of glucose as glycogen by the liver. Glucose on the other hand, which is present beside fructose in honey, enhances the absorption of fructose and promotes its hepatic actions through its enhanced delivery to the liver [45, 46].

The pancreas is an important organ in diabetes, because it secrets two glucose-regulating hormones—insulin and glucagon—and honey might protect this organ against oxidative stress and damage with its antioxidant molecules, this being another potential mechanism of hypoglycemic effect of honey [32, 47].

Different studies were made on the effect of fructose on glycemic control, glucose-regulating hormones, appetite-regulating hormones, body weight, food intake, and oxidation of carbohydrates or energy expenditure [38, 44, 48–61].

Fructose administrated alone or as part of sucrose molecule in normal rats improved glucose homeostasis and insulin response compared to rats which received glucose [62]. Other studies show that fructose supplementation in normal or type 2 model of diabetic rats produced lower levels of plasma insulin and glucose, more than other administrated sugars [38].

## 3. Animal Model Experiments

Different animal models were used to study the possible hypoglycemic effect of honey. The most used experimental tool for inducing type 1 and type 2 diabetes is streptozotocin and alloxan of appropriate doses [63–66].

A study of six weeks [67] on healthy nondiabetic rats fed with a honey-containing diet exhibits good results: weight was reduced statistically significant, but no significant decreasing for glycosylated hemoglobin or food intake was observed.

Long-term honey feeding in Sprague-Dawley rats (52 weeks) produces a significant decrease of HbA1c levels but increases HDL cholesterol [68]. In sucrose-fed and sugar-free diet-fed rats, in the same experiment, HDL cholesterol levels were decreased and no other differences were observed for other lipids. Weight gain was similar for honey and sugar-free diet-fed rats but less compared to sucrose-fed rats.

Busseroles et al. [69] fed healthy rats with 65 g/100 g combined fructose and glucose or a honey-based diet for two weeks and the level of blood fructose, serum vitamin E, and serum vitamin E/triglycerides increased, while glucose content remains unchanged and triglyceride content decreased.

Feeding healthy rats with a diet containing 20% honey for 33 days, Nemoseck et al. [70] obtained significant decrease of triglycerides, leptin content, body weight, food/energy intake, and epididymal fat weight but not significantly glucose decrease, total cholesterol decrease, adiponectin, and C-reactive proteins. This experiment shows that longer period of feeding must be used, to obtain significant results.

Erejuwa et al. [11, 47] found no significant differences in fasting blood glucose or body weight in honey-fed rats.

If honey was demonstrated to have hypoglycemic effect in healthy animals, the same beneficial effect was observed in induced diabetic animals. A very important observation regarding honey and diabetes is that honey augments the antihyperglycemic effect of standard antidiabetic drugs in induced diabetes [10, 33].

Rabbits with diabetes induced by alloxan were used in one experiment, and three types of sweeteners were used for feeding the animals [65]. Pure honey of *Apis florea* and *Apis dorsata* and adulterated honey were given in different doses in a rabbit's diet, and a dose-dependent rise in blood glucose was registered.

Another study [66] of alloxan-induced diabetic rats fed with honey and healthy rats fed with fructose shows different results: glucose decreased significantly in alloxan-induced diabetic rats and not significantly in fructose-fed rats. Body weight increased in healthy fructose-fed rats, and hypoglycemic effect and also the same effect were found for streptozotocin-induced diabetic rats [71].  Table 1 summarizes the preclinical studies on healthy and induced diabetic animals, using honey solution or other sweeteners in their diet.

## 4. Honey versus Sugars in Human Clinical Trials

Human diet must have all types of nutrients required in the metabolic transformations and life support. Water, proteins, lipids, carbohydrates, vitamins, minerals, amino acids, and bioactive compounds are needed by the human body, and all of these compounds are taken from the diet. Maintaining a healthy life, equilibrate diet, and intake of each and every one of these nutrients is the key factor of health in general. Different diseases have as a starting point unbalances in metabolism, because of lack or excess of one or more nutrients.

Diabetes, as stated before, represents the high level of blood sugars due to low or no insulin production in the body. Experimental studies on animals suggest the beneficial effects of honey as a diet supplement and encouraging results on control of diabetes mellitus and additional complications are presented in medical studies; the experiments and reports on humans (healthy or diabetic) are rather sparse.

The published studies present favourable effects of honey in both healthy and diabetic subjects [16, 31, 72–76]. Since oxidative stress is implicated and mainly responsible for diabetes development, the antioxidant effects of honey are very important in this disease management [77].

The study of Al-Waili [78] on healthy, diabetic, or patients with hypertriglyceridemia shows promising results, when honey was used in their diet, compared with dextrose and sucrose. Thus, lipid profile was improved, normal and elevated C-reactive protein was lowered, and also homocysteine value and triacylglycerol were decreased in patients with hypertriglyceridemia. In diabetic patients, honey compared with dextrose caused a significantly lower rise of plasma glucose level (PGL). Honey caused greater elevation of insulin compared to sucrose; after different time of consumption, it reduces blood lipids, homocysteine, and CRP in normal subjects. The conclusion was that honey compared with dextrose and sucrose caused lower elevation of PGL in diabetics. This experimental study on healthy, diabetic, and hyperlipidemic human subjects demonstrates the different intake rate of refined sugar and honey, the raising of blood sugar and also raising their insulin levels.

Sugar is a refined product, obtained from different natural sources, but follows a technological process, leading to an almost pure substance—sucrose—highly used in modern life in the food industry.

Honey, on the other hand, being also a natural sweet product, has a complex composition, but compared to sugar, it has a lower glycemic index and energetic value. When we talk about refined sugar, it is easy to state the exact chemical composition, very simple actually, but talking about honey, many aspects should be considered regarding its composition. Botanical and geographical origins determine the specific composition and properties of all types of honeys.

Table 2 presents comparatively the chemical composition of refined sugar and honey.

The fact that refined sugar is almost 100% sucrose, and very small amounts of other components compared to honey, makes the last one, an important sweetener, with almost 80% simple sugars from the total chemical composition (35–40% fructose and 30–35% glucose).

Even though the exact mechanism by which honey may have beneficial effects upon blood glucose is not very clear; from comparative experiments, some conclusions about the importance of fructose in honey are available. Fructose is known to stimulate glucokinase in hepatocytes, which plays an important role in the uptake and storage of glucose as glycogen by the liver [79], the amount of fructose in honey being very important for its hypoglycemic effects.

A study on humans [80] evaluated for a large period of time wherein a group of twenty adult patients with type 2 diabetes volunteered to stop their medication and use honey as treatment for their disease. This nonrandomized, open clinical trial aiming to study the safety and efficiency of honey as unique treatment revealed interesting results (Table 3).

Besides glycemic index (GI), peak incremental index (PII) is used to assess the glycemic effect (the effect on blood glucose level after ingestion of various foods) [81].

C-peptide is considered a good marker of insulin secretion, being cosecreted with insulin by the pancreatic cells as a by-product, with no biological activity of its own [82], of the enzymatic cleavage of proinsulin to insulin. Scientific studies regarding the effects of honey on insulin and C-peptide levels are controversial in healthy and diabetic patients [54, 83, 84].

A study made in the National Institute of Diabetes in Cairo, Egypt, on twenty diabetic young patients and ten healthy nondiabetic ones try to elucidate this controversy [73]. Glucose, sucrose, and honey were administrated diluted with 200 ml water, according to the patient's weight (amount of sugar/honey = weight of the subject in kg × 1.75, with a maximum of 75 g). The diluted sugars and honey were ingested in the morning by every participant, one week apart for each sugar type, the whole test lasting for three weeks. Blood tests were made before ingestion and after every 30 min postprandial of sugars, until 120 min (2 hours). Serum C-peptide level and glucose assay were measured for all blood samples.

The glycemic index and peak incremental index were lower both in patients and control group, when honey was used compared to glucose and fructose, but the level of C-peptide was different in patients and control group.

Honey causes a postprandial rise of plasma C-peptide levels compared to sucrose and glucose in nondiabetic patients, suggesting that honey might have a direct stimulatory effect on the healthy beta cells of the pancreas [73].

Although honey has lower GI than sugar (Table 2), an average value for honey is presented [85], according to fructose/glucose ratio, and GI value of different honeys is also different [86].

Twenty healthy subjects from Erciyes University, Kayseri, Turkey, were subjected voluntarily to a test of ingesting 50 g of pure glucose in 250 ml water and an amount of honey that corresponds to 50 g glucose (accordingly to the physicochemical analysis of honey used in the test). Capillary blood samples were taken from the finger in the next morning after sugar consumption and again every 15 minutes after second ingestion of sugars in the next day, until 120 minutes. Serum glucose and serum insulin level decreased after 2 hours of honey intake, and C-peptide level increased slightly 2 hours after honey intake. This study demonstrates how different types of honey, having different GI values, influence the parameters usually measured for diabetes control in a different manner [85].

Sixty healthy subjects aged 18 to 30 years, enrolled in one experiment in Isfahan University of Medical Science, Iran [87], receive 80 g of honey and 80 g sucrose dissolved in 250 ml water once a day for six weeks. Systolic blood pressure (SBP), diastolic blood pressure (DBP), and fasting blood sugar (FBS) were determined from each participant at the beginning and in the end of the study. No significant change was registered in SBP and DBP in both groups at the beginning and in the end of the study, but FBS registered a significant reduction in the honey group at the end of the study, compared to the sucrose group [87].

Different studies mentioned before show that honey consumption reduces body weight but also blood glucose in healthy and diabetic patients compared to sugar intake. A study on type 2 diabetic patients consuming natural honey shows that body weight may be reduced and blood lipids and glucose as well [31]. The study consists of 58 patients with type 2 diabetes, with fasting blood sugar of 110–220 mg/dl, with same oral hypoglycemic drugs, but no insulin treatment. The experimental group (*n* = 25) receives natural honey for eight weeks following an experimental scheme, and the control group (*n* = 23) did not receive honey or other sweeteners. The participants continued their usual diet over the study period. The body weight and fast blood sugar were measured every 2 weeks, and constant decreasing was registered [31]. Scientific studies reviewed by Erejuwa et al. [12, 33] demonstrate that fructose and oligosaccharides from honey contribute to its hypoglycemic effect. In addition to lowering oxidative stress and hyperglycemia, honey consumption ameliorates other metabolic disorders associated with diabetes, such as reduced levels of hepatic transaminases, triglycerides, and glycosylated hemoglobin (HbA1c) and increased HDL cholesterol [12, 31].

Several honey types from different parts of the world ameliorate metabolic abnormalities in type 1 and type 2 diabetic patients [36, 73, 88]. These studies investigate the acute effects of honey on hyperglycemia and metabolic disorders, because the diabetic parameters were measured postprandial in studies which last from two to eight weeks. Table 3 summarizes the clinical studies on humans, applied treatment, and the main obtained results.

## 5. Honey in Diabetic Wound Healing

Besides the health benefits of ingesting honey in diabetes, another important use of honey could be in managing diabetic wounds [89]. These wounds are not like typical wounds, they are slower in healing or they do not heal at all, leading to complications that conventional medications do not work.

Honey was used in alternative medicine for healing different wounds since ancient times, the use of honey in diabetic wound management being more recent. Diabetic patients sometimes suffer from different complications such as arterial disease, vascular problems, ulcerations, and foot complications [90, 91].

Even if diabetic wounds are similar to wounds from normal patients, the healing process in the former is very slow and problematic and the medical costs are extremely high. Honey is a potential candidate to be used in these treatments because it is available, natural, and not expensive. But how can honey work at the wound site? The honey diluted with water or different body fluids forms hydroxyl radicals and hypochlorite anions at the wound site. The antioxidants present in the honey act through two different mechanisms in a wound: first, antioxidants fight against microorganisms and lower the infection in the wound [75, 92, 93]; second, the same antioxidants reduce the reactive oxygen species and inflammation caused by the wound, helping in the healing process [94–96].

The antimicrobial activity of honey is due to acidic pH, osmotic effect, hydrogen peroxide, and nitric oxide. The presence of nitric oxide metabolites in honey as well as the production of NO products by honey in different body fluids improves the healing process [74, 80, 97].

Debridement, wound odor, scar formation, and inflammation control are very important in diabetic wound management [89]. The slow healing process in diabetic wounds is due to the peripheral arterial diseases and peripheral neuropathy that occur with diabetes; the blood vessels tend to shrink, reducing blood circulation in the respective areas. The nerves do not receive enough blood (nutrients) and may become damaged and more vulnerable to injury. The stimulating tissue growth when honey is used is due to the chemical composition, the presence of assimilable sugars, vitamins, amino acids, and phenolics that increases oxygen and nutrients in the wound area [98, 99].

Numerous studies show evidence of successful honey treatments against diabetic wounds all over the world [100–105]. Honey applications reduce wound ulcer pain and size and deodorization of the wound, and reduction of healing time and are safe and there are no side effects.

A recent study [106] brings new evidence in demonstrating the effects of Manuka honey in wound healing. The results reported by the authors, based on the capacity of this type of honey to improve the responsiveness to oxidative damage, as well as stimulation of cell proliferation, could help to understand how Manuka honey develops its healing effect on wounds.

Although, some guidelines for honey applications must be used such as natural unheated honey should be used in treatments and stored in dark glass bottles in cool places. Different medical grade honey with standardized antibacterial activity for use in wound treatments are known, such as Apiban (Apimed: Cambridge, New Zealand), Woundcare 18+ (Comvita: Te Puke, New Zealand), and Medihoney (Capilano: Richmonds, Queensland, Australia) [99]. If these honeys are not available, any dark honey with high antibacterial activity may be used.

## 6. Conclusions

Considerable evidence from experimental studies shows that the honey may provide benefits in the management of diabetes mellitus. The benefits could be a better control of the hyperglycemic state, limiting other metabolic disorders and diminishing the deleterious effects on different organs that may produce diabetic complications. Anyway, there are some data and literature with contrary discussions regarding the use of honey in diabetic diseases.

Animal models of diabetes were employed chemically (streptozotocin or alloxan), and this may not entirely reflect the development of type 2 diabetes in humans. More studies on animals are necessary but following other animal models, closer than human type 2 diabetes.

Optimal doses for human consumption must be established, and longer period experiments must be developed, due to the fact that diabetes mellitus is a chronic disease.

Answering the main question of the study, it is true that honey may be used as a potential antidiabetic agent that has the potential to reduce the complications of diabetes, long-term studies using honey as an alternative or a complementary therapy in human subjects suffering from type 2 diabetes mellitus are needed, with a larger number of patients, randomized clinical trials set up with different levels of diabetes, treated with different doses of honey, following both short-term and long-term treatment.

As stated recently [107], “The use of honey in diabetic patients still has obstacles and challenges and needs more large sample sized, multicenter clinical controlled studies to reach better conclusions.”

## Figures and Tables

**Table 1:** Preclinical studies on animal models regarding the effect of honey on induced diabetes mellitus.

Ref.	Animal models	Applied treatment	Obtained results
[10]	60 diabetic rats divided into 6 groups: (1) distilled water, (2) honey, (3) glibenclamide, (4) glibenclamide and honey, (5) metformin, and (6) metformin and honey	Distilled water, honey, glibenclamide, glibenclamide and honey, and metformin or metformin and honey treatment orally once a day for 4 weeks	Honey significantly increased insulin (0.41 ± 0.06 ng/ml), decreased hyperglycemia (12.3 ± 3.1 mmol/L), and fructosamine (304.5 ± 10.1 *μ*mol/L). Glibenclamide and metformin alone significantly reduced hyperglycemia, but combined with honey, produced significantly much lower blood glucose (8.8 ± 2.9 or 9.9 ± 3.3 mmol/L, resp.) compared to glibenclamide or metformin alone (13.9 ± 3.4 or 13.2 ± 2.9 mmol/L).

[11]	Diabetic rats (6 rats/group) induced by streptozotocin (STZ) 60 mg/kg	Distilled water (0.5 ml/day) Honey (0.2 g/kg/day, 1.2 g/kg/day, and 2.4 g/kg/day) oral gavage for 4 weeks	Total antioxidant status (TAS), activities of catalase (CAT), glutathione peroxidase (GPx), glutathione reductase (GR), and glutathione-S-transferase (GST) were significantly reduced, while superoxide dismutase (SOD) activity was upregulated in kidneys of diabetic rats. Lipid peroxidation (TBARS) and fasting plasma glucose (FPG) were significantly elevated while body weight was reduced in diabetic rats. Honey significantly increased body weight, TAS, and activities of CAT, GPx, GR, and GST in diabetic rats.

[12]	Adult male Sprague-Dawley rats; diabetes induced by STZ (60 mg/kg body weight)	Tualang honey (1.0 g/kg body weight)	Tualang honey supplementation in diabetic rats reduces elevated levels of AST and ALT and also produces a hepatoprotective effect in STZ-induced diabetic rats.

[14]	6 groups of 6 rats/group	(1) Control rats feed with standard pellet diet and water; (2) diabetic rats as untreated diabetic control; (3) diabetic rats treated with honey 1.0 g/kg BW for 21 days; (4) hyper cholesterol rats: cholesterol (1.5%) and cholic acid (0.5%) mix with diet; (5) hyper cholesterol rats treated with honey (1.0 g/kg BW for 21 days); and (6) diabetic rats treated with glibenclamide (0.5 mg/kg)	Honeybee treatment significantly decreases blood glucose level in diabetic rats. TC, TG, LDL, and VLDL are significantly decreased whereas HDL significantly increases. The SGPT, SGOT, and CRP were significantly decreased.

[33]	8 groups of diabetic rats (5–7 animals/group)	Treatments/groups: (1) distilled water (0.5 ml); (2) honey (1.0 g/kg); (3) metformin (100 mg/kg); (4) metformin and honey; (5) glibenclamide (0.6 mg/kg); (6) glibenclamide and honey; (7) metformin and glibenclamide; and (8) metformin, glibenclamide, and honey orally, once a day for 4 weeks	Malondialdehyde (MDA) levels, glutathione peroxidase (GPx), and superoxide dismutase (SOD) activities were significantly elevated while catalase (CAT) activity, total antioxidant status (TAS), reduced glutathione (GSH), and GSH : oxidized glutathione (GSSG) ratio were significantly reduced in the diabetic kidneys. CAT, glutathione reductase (GR), TAS, and GSH remained significantly reduced in the diabetic rats treated with metformin and/or glibenclamide. In contrast, metformin or glibenclamide combined with honey significantly increased CAT, GR, TAS, and GSH.

[47]	Diabetic (2 groups) and nondiabetic rats (2 groups)	Diabetic rats were administered distilled water (0.5 ml/d) and Tualang honey (1.0 g/kg/d). Nondiabetic rats received also distilled water (0.5 ml/d) and Tualang honey (1.0 g/kg/d)	The honey-treated diabetic rats had significantly reduced blood glucose levels [8.8 (5.8) mmol/L; median (interquartile range)] compared with the diabetic control rats [17.9 (2.6) mmol/L].

[65]	8 groups of rabbits (6 animals/group); groups I to IV were normal and healthy (nondiabetic) and groups V to VIII were diabetic induced by alloxan monohydrate	Group I: untreated control received 20 ml of water orally. Groups II–IV treated orally with 5, 10, and 15 mg/kg BW honey diluted up to 20 ml/kg with distilled water. Groups V–VI treated with tolbutamide (250 mg and 500 mg). Group V: diabetic control, treated with 20 ml of water. Groups VI–VIII treated orally with 5, 10, and 15 ml/kg BW of honey diluted to 20 ml with distilled water	Oral administration of pure honeys in 5 ml/kg/doses could not produce a significant (*P* > 0.05) increase in glucose levels in normal and alloxan-diabetic rabbits whereas the adulterated honey significantly raised the blood glucose levels in normal and hyperglycemic rabbits even at this low dosage.

[66]	48 matured male Wistar rats separated into 6 groups	Group 1a: control had standard rat chow for 3 weeks. Group 1b: fed with honey along with standard rat chow for 3 weeks. Group 2a: alloxan-induced diabetes and standard rat chow for 3 weeks. Group 2b: alloxan-induced diabetes, fed with honey and standard rat chow. Group 3a: standard rat chow and fructose for 3 weeks. Group 3b: standard rat chow fructose for three weeks than honey along with standard rat chow and fructose for 3 weeks	At the end of three weeks, it was found that daily ingestion of honey for 3 weeks progressively and effectively reduced blood glucose level in rats with alloxan-induced diabetes. Honey also caused a reduction in hyperglycemia induced by long-term ingestion of fructose, albeit to a lesser degree than its effect on alloxan-induced hyperglycemia. Honey could not reduce blood glucose in controlled rats that received neither alloxan treatment nor fructose ingestion, even though it caused an increase in body weight, irrespective of other substances concomitantly administered to the rats.

[67]	40 six-week-old Sprague-Dawley rats	A powdered diet that was either sugar-free or which contained 8% sucrose, 8% mixed sugars as in honey, or 10% honey freely for 6 weeks	HbA1c and triglyceride levels were significantly higher in all sugar treatments compared with rats fed with a sugar-free diet.

[68]	55 Sprague-Dawley rats aged approximately 8 weeks	3 experimental diets were prepared to contain no sugar, 7.9% sucrose, or 10% honeydew honey	Weight gain was substantially reduced in honey-fed rats compared with those given a sucrose-based diet; the finding that consuming honey increases HDL cholesterol levels is still a significant result though. There have been strong associations seen between low HDL cholesterol levels and the increased risk of cardiovascular disease.

[71]	36 rats divided into 6 groups of 6 animals. Diabetes was induced by STZ (60 mg/kg; ip)	Diabetic rats received distilled water (0.5 ml/day), honey (1.0 g/kg/day), and metformin (100 mg/kg/day) or a combination of metformin (100 mg/kg/day) and honey (1.0 g/kg/day) orally for four weeks. Similarly, two groups of nondiabetic rats received distilled water (0.5 ml/day) and honey (1.0 g/kg/day)	Honey significantly increased GSH, TAS, and activities of CAT and GR in diabetic rats while FPG, MDA levels, and SOD activity were decreased. The final results indicate that honey exerts hypoglycemic effect and ameliorates renal oxidative stress.

**Table 2:** Average chemical composition of honey compared to sugar.

Component/100 g	Honey^∗^	Sugar
Glycemic index	58	60
Calories	300 Kcal	387 Kcal
Sugars	80.0 g	99.9 g
Lipids	0.02 g	—
Protein	0.3 g	—
Calcium	6.0 mg	1.0 mg
Iron	0.42 mg	0.01 mg
Magnesium	2.0 mg	—
Phosphorus	4.0 mg	—
Zinc	0.22 mg	—
Potassium	52.0 mg	2.0 mg
Vitamin C	0.5 mg	—
Vitamin B2	0.038 mg	0.019 mg
Vitamin B3	0.121 mg	—
Vitamin B5	0.068 mg	—
Vitamin B6	0.024 mg	—
Vitamin B9	2.0 *μ*g	—
Water	17.0 g	0.03 g

^∗^Values specified for honey represent an average of floral and honeydew honey.

**Table 3:** Clinical studies regarding the effect of honey in human diabetic subjects.

Ref	Research groups	Honey/sugars treatment schemes	Obtained results
[16]	17 subjects (control group) 38 subjects (experimental group)	70 g sucrose daily for 30 days in the control group and 70 g of honey in the experimental group	Honey caused a mild reduction in body weight (1.3%) and body fat (1.1%), reduced total cholesterol (3%), LDL-C (5.8), triacylglycerol (11%), FBG (4.2%), and CRP (3.2%), and increase HDL-C (3.3%) in normal subject and in patients honey cause reduction in total cholesterol by 3.3%, LDL-C by 4.3%, triacylglycerol by 19%, and CRP by 3.3%.

[31]	48 type II diabetic patients: Honey group Control group	1 g/kg BW/day for 2 weeks; 1.5 g/kg BW/day for next 2 weeks; 2 g/kg BW/day for next 2 weeks; and 2.5 g/kg BW/day for the last 2 weeks	Body weight, total cholesterol, low-density lipoprotein cholesterol, and triglyceride decreased, and high-density lipoprotein cholesterol increased significantly in the honey group. The levels of hemoglobin A (1C) increased significantly in the honey group.

[36]	24 healthy subjects, 16 type II diabetic subjects 6 patients with hypertension	12 healthy subjects receive inhalation with distilled water for 10 min; after one week, they received inhalation of honey solution (60% wt/*v*) for 10 min. 12 healthy subjects received inhalation of 10% dextrose for 10 min	Honey inhalation significantly reduced random blood glucose level from 199+/−40.9 mg/dl to 156+/−52.3 mg/dl after 30 min. Fasting blood glucose level was reduced after honey inhalation during 3 hr postinhalation, which was significant at hour 3. Intensity of hyperglycemia was significantly lowered in glucose tolerance test when patients received honey inhalation.

[54]	32 type II (noninsulin-dependent) diabetic patients	Diet of 25 g glucose, fructose, or lactose or 30 g honey, 50 g white bread, 125 g white rice or potatoes, and 150 g apples or 260 g carrots	Blood glucose and plasma insulin were measured at zero time and then at 15, 30, 60, 90, and 120 min after the meal. Counting the blood glucose increase after glucose as 100%, the corresponding increases in glycemia for other carbohydrates were fructose, 81.3%; lactose, 68.6%; apples, 46.9%; potatoes, 41.4%; bread, 36.3%; rice, 33.8%; honey, 32.4%; and carrots, 16.1%.

[73]	20 young type I diabetic patients in the experimental group; 10 healthy nondiabetics in the control group	Calculated amount of glucose, sucrose, and honey (amount = weight of the subject in kg × 1.75 with a maximum of 75 g/patient)	Honey, compared to sucrose, had lower GI and PII in both patients and control groups. In the patient group, the increase in the level of C-peptide after using honey was not significant when compared with glucose or sucrose.

[76]	30 individuals with a proven parental (mother or father) history of type II diabetes mellitus	Glucose diet supplementation Honey diet supplementation	The plasma glucose levels in response to honey peaked at 30–60 minutes and showed a rapid decline as compared to that of glucose. Significantly, the high degree of tolerance to honey was recorded in subjects with diabetes as well, indicating a lower glycemic index of honey.

[78]	48 subjects: healthy and diabetic and with hyperlipidemia	(i) Dextrose solution (250 ml of water containing 75 g of dextrose) or honey solution (250 ml of water containing 75 g of natural honey) (ii) Dextrose, honey, or artificial honey (250 ml of water containing 35 g of dextrose and 40 g of fructose) (iii) Honey solution, administered for 15 days (iv) Honey or artificial honey (v) 70 g of dextrose or 90 g of honey in patients with type 2 diabetes mellitus (vi) 30 g of sucrose or 30 g of honey in diabetic patients	Healthy subjects: dextrose elevated PGL at 1 and 2 hours and decreased PGL after 3 hours. Honey elevated PGL after 1 hour and decreased it after 3 hours. Elevation of insulin and C-peptide was significantly higher after dextrose than after honey. Dextrose slightly reduced cholesterol and low-density lipoprotein cholesterol (LDL-C) after 1 hour and significantly after 2 hours and increased TG after 1, 2, and 3 hours. Artificial honey slightly decreased cholesterol and LDL-C and elevated TG. Honey reduced cholesterol, LDL-C, and TG and slightly elevated high-density lipoprotein cholesterol (HDL-C). Honey consumed for 15 days decreased cholesterol, LDL-C, TG, CRP, homocysteine, and PGL but increased HDL-C. Hypertriglyceridemic patients: artificial honey increased TG, but honey decreased TG. In patients with hyperlipidemia, artificial honey increased LDL-C, while honey decreased LDL-C. Honey decreased cholesterol, LDL-C, and CRP after 15 days. In diabetic patients, honey compared with dextrose caused a significantly lower rise of PGL. Elevation of PGL was greater after honey than after sucrose at 30 minutes and was lower after honey than it was after sucrose at different intervals. Honey caused elevation of insulin compared to sucrose after different intervals and lower elevation of PGL in diabetics.

[80]	20 adult patient volunteers suffering from type 2 DM and its associated metabolic disorders from 30 to 65 years and both sexes	Honey dose of 2 g/kg BW/day, (i) 50 ml (60 g) honey was dissolved in water (ratio of 1 : 3) and given before meals twice daily; (ii) the remaining 25 ml (30 g) was used for sweetening purposes	Honey consumption resulted in more hyperglycemia in these patients but without diabetic ketoacidosis (DKA) or hyperglycemic hyperosmolar state (HHS). Longer-term honey consumption resulted also in weight reduction in all the patients, and control of the blood pressure in the patients, who had hypertension before the honey intervention. The cardiovascular status improved in the patients, who had coronary heart disease (CHD) before the intervention.

[88]	50 patients with type I diabetes mellitus 30 controls without diabetes	The honey dose: 1.75 g/kg BW Sucrose dose: 1.75 g sugar/kg BW	The GI and PII of either sucrose or honey did not differ significantly between patients and controls. Both the GI and PII of honey were significantly lower when compared with sucrose in patients and controls. In both patients with diabetes and controls, the increase in the level of C-peptide after the honey was significant when compared with either glucose or sucrose.

## Abbreviations

ALT: Alanine aminotransferase
AST: Aspartate aminotransferase
BW: Body weight
CAT: Catalase
CHD: Coronary heart disease
CRP: C-reactive protein
DBP: Diastolic blood pressure
DKA: Diabetic ketoacidosis
DM: Diabetes mellitus
FBG: Fasting blood glucose
FBS: Fasting blood sugar
FPG: Fasting plasma glucose
GI: Glycemic index
GPx: Glutathione peroxidase
GR: Glutathione reductase
GSH: Reduced glutathione
GSSP: Oxidized glutathione
GST: Glutathione-S-transferase
HbA1C: Glycated hemoglobin
HDL: High-density lipoproteins
HHS: Hyperglycemic hyperosmolar state
MDA: Malondialdehyde
NO: Nitric oxide
PII: Peak incremental index
PGL: Plasma glucose level
SBP: Systolic blood pressure
SGOT: Serum glutamic oxaloacetic transaminase
SGPT: Serum glutamate pyruvate transaminase
SOD: Superoxide dismutase
STZ: Streptozotocin
TAS: Total antioxidant status
TBARS: Thiobarbituric acid reactive substances
TC: Total cholesterol
TG: Triglyceride
VLDL: Very low-density lipoprotein.
